# The Oxygen Consumption Kinetics of Commercial Oenological Tannins in Model Wine Solution and Chianti Red Wine

**DOI:** 10.3390/molecules25051215

**Published:** 2020-03-08

**Authors:** Jelena Jeremic, Isara Vongluanngam, Arianna Ricci, Giuseppina Paola Parpinello, Andrea Versari

**Affiliations:** 1 Department of Agricultural and Food Sciences, University of Bologna, Piazza Goidanich 60, 47521 Cesena (FC), Italy; jelena.jeremic4@unibo.it (J.J.); giusi.parpinello@unibo.it (G.P.P.); andrea.versari@unibo.it (A.V.); 2 RUFFINO S.r.l.—a Constellation Brands Company—Winemaker, P.le Ruffino 1, 50065 Pontassieve (FI), Italy; isara.vongluanngam@gmail.com; 3 Ecole Supérieure d’Agriculture, 55 Rue Rabelais, 49000 Angers, France

**Keywords:** antioxidants, oxygen consumption rate, Sangiovese, sustainable winemaking, tannins

## Abstract

One property of oenological tannins, oxygen reactivity, is commonly exploited in winemaking. The reactivity is mediated by the presence of catalysts (i.e., transition metals and sulfur dioxide) and protects wine against oxidation. This work compares the oxygen consumption rate (OCR) of four commercial oenological tannins (two procyanidins from grape skin and seed, an ellagitannin from oak wood and a gallotannin from gallnut) in a model wine solution and Chianti red wine. All samples were subjected to consecutive cycles of air saturation at 20 °C to increase the total level of oxygen provided. After each cycle, the oxygen level was measured by means of a non-invasive luminescent sensor glued to a transparent surface (sensor dots) until there was no further change in substrate reactivity. The OCR followed first-order kinetics, regardless of the tannin. As expected, the ellagitannin showed the fastest OCR, followed by the two from grape seeds and skins and finally the gallotannin. The total O_2_ consumption in the red wine was almost double that of the model solution, due to the oxidation of wine substrates. The measurement of OCR is helpful for setting up an advanced winemaking protocol that makes use of tannins to reduce the use of sulfur dioxide.

## 1. Introduction

Managing oxygen concentration is a big challenge in winemaking because the oxygen level affects wine development in terms of sensory and chemical characteristics. Moderate, controlled exposure to oxygen by means of winemaking practices like micro-oxygenation [1,2] seems to enhance wine quality during aging, while uncontrolled oxygen exposure—too much or too little—can lead to the creation of reductive off-flavors or oxidative spoilage of wine, respectively [3,4,5,6].

Currently, sulfur dioxide is the main antioxidant used to protect wine against the detrimental influence of oxygen. However, concerns about its allergenic side effects have created a search for effective alternatives [7,8], such as oenological tannins. Tannins have various health benefits, including antioxidant, antitumor, cardioprotective, anti-inflammatory and antimicrobial activity [9]. Grape tannins in particular seem to be effective dietary supplements [10].

Chemically, tannins (i.e., proanthocyanidins) are high molecular weight (Mw > 500), naturally occurring phenolic compounds which precipitate protein. Their use is authorized in winemaking by the International Organization de la Vigne et du Vin (OIV 2017) as ‘processing aids’ to prevent protein instability and iron haze in musts and wines [11]. Although the OIV does not mention tannins’ antioxidant properties, it continues to update its resolutions to consider the multiple technological opportunities offered by tannins in winemaking. Going forward, tannins must be properly evaluated to maximize their positive effects—without producing undesired side effects. Recent studies have reported the following advantages of oenological tannins: (i) protection against chemical and enzymatic oxidation [1], (ii) improved stability of wine color through co-pigmentation [12] and the formation of polymeric pigments [13] and (iii) improved mouthfeel due to a modulation of the perceptions of astringency and bitterness in wine [14,15].

When astringency intensity was compared for model solutions of various tannin extracts at 0.5 g/L, the quebracho tannin was the most astringent, followed (in descending order) by gall, chestnut, oak, grape skin, grape seed and tara [16]. In terms of redox reactions, gallotannins have superior radical scavenging capacity, the ellagic ones are able to chelate iron(II) strongly and the condensed tannins have a significant ability to scavenge peroxyl radicals [17].

The current understanding of wine oxidation involves the preliminary reaction between oxygen with ferrous iron to form hydrogen peroxide and ferric iron; the latter reacts with phenolic compounds to regenerate the iron(II) and produce a quinone. Several radical species can then be formed, depending on the reaction conditions [18,19]. Thus, the role of phenolic compounds in wine oxidation seems primarily linked to hydrogen peroxide and a radical-quenching reaction—and eventually to metal chelation. Notably, the quinone is able to regenerate the original polyphenol, which then has the capacity to absorb another equivalent of oxidation, therefore affecting the rate of oxygen consumption [2]. The oxidation of condensed tannins, gallo- and ellagitannins was previously investigated by means of electrochemical oxidation to establish that only procyanidin–quinone oxidation products recycle back to procyanidin [20].

Further complicating attempts to understand oxidation in wine is the fact that acidity is also an important factor. Total oxygen uptake is greater in acidic conditions than in alkaline conditions, probably because in acidic conditions, oxidizable phenols are converted to quinones more slowly, so that the generated quinones have more time to create polymers with the remaining phenols [21].

However, the relationship between the degree of polymerization and the antioxidant activity of proanthocyanidins is largely unknown. The flavonoids with the most hydroxyl groups are most easily oxidized; for simple flavonoid oligomers, the degree of polymerization seems correlated with the ability to scavenge free radicals [22]. This relationship seems to reach a plateau for condensed tannin at about 9–10 units, which is considered the level at which the activity of proanthocyanidins becomes significant [23,24].

The oxygen consumption rate (OCR) of tannins depends on their chemical structure [5] and on the number of oxygen saturation cycles performed on the wine; the initial saturations seem to have the fastest oxygen consumption rate [4]. In 1977, Perscheid and Zurn reported that a Muller–Thurgau juice did not demonstrate decreased oxidative capacity until it had been saturated up to 40 times [25]. In contrast, Amano et al. (1979) reported reduced oxidation after the first saturation: by adding fresh phenolic substrates and noting a renewed increase in oxygen consumption they proved that the drop was due to the depletion of substrate [26].

Sangiovese’ is the most widespread *Vitis vinifera* red grape cultivar in Italy, producing the famous Chianti and Brunello di Montalcino wines in Tuscany [27] and previous studies on the effects of oenotannins focused on stabilizing wine color [13] and evaluating sensory [15]. However, if tannins are to replace (or reduce the use of) sulfur dioxide in wine, there is a need for further information on the effect of oenological tannins on oxygen consumption.

This study tests the antioxidant ability of two condensed and two hydrolysable oenological tannins, comparing their oxygen consumption rates in a model solution and commercial Chianti red wine.

## 2. Results and Discussion

The modulation of oxygen exposure during winemaking and fining is a complex landmark, affected by the extent of oxygen exposure, aeration conditions and wine composition. Due to the complexity of factors involved it is difficult to set the best level of oxygen exposure; at the current state of art, the application of consecutive cycles of oxygen enrichment/consumption is considered a suitable approach to simulate winery conditions and forced oxidation levels [28,29,30].

The enological tannins used in this experiment were from commercial sources and effectively used at winemaking level. Their composition was assessed with reliable analytical protocols (see Materials and Methods section) and the little amount of tannins (i.e., polymers with ≥3 units) compared to total polyphenolics (Table 1) highlighted the large presence of low molecular weight phenolics (up to dimers), which is confirmed by literature, together with the low amount of tannins in the wood [31,32,33]. According to Harbertson et al. (2012), gallotannins and quercus hydrolysable tannins contain 12% and 27% of tannins, respectively [14]. Similarly, Vignault et al. (2018) found that tannins range from 16.1 ± 1.7% to 98.7 ± 4.6%, which indicates the existence of a wide variability among oenological tannins [34].

**Figure 1:**
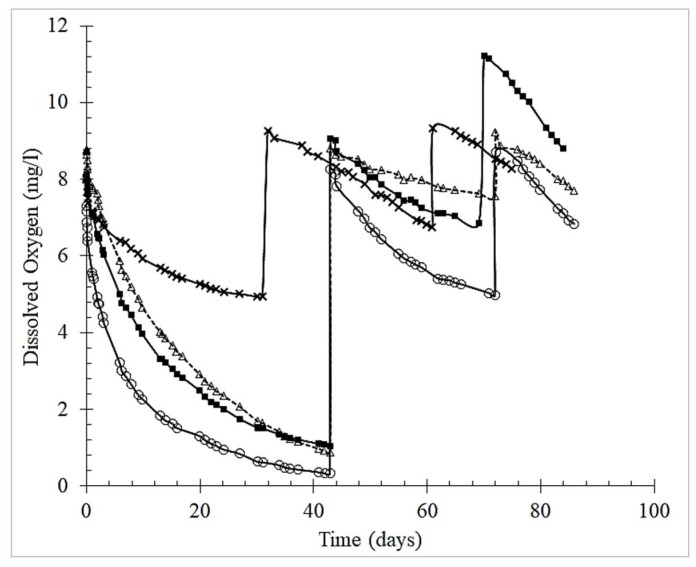
Oxygen consumption over time in the model solution containing tannins, for three consecutive saturations. Legend: (Δ) grape seed tannin; (■) grape skins tannin; (○) ellagitannin; (x) gallotannin.

The O_2_ measurements were carried out through up to four saturation cycles for about 90 days (36 trials, including model solution and red wine). The duration of the monitoring is compatible with the production of young red wines [35] and the dosage of tannin added to model wine solution is consistent with the literature [36,37]. Figure 1 shows the oxygen consumption over time in the model solution for three consecutive saturations, demonstrating the dose-response effect of oenological tannins. During the first 30 days the Total Packaging Oxygen (TPO) consumed by model solutions with tannins at 1 g/L fully consumed the first oxygen saturation (>6.96 mg O_2_/L), except gallotannins (3.10 mg O_2_/L). Noteworthy, the model wine with gallotannin showed in the third saturation an increase of approx. 20% in the level of oxygen saturation. This unexpected value might be due to lack of HSO removal during the third saturation (see material and method for details) although the reflection of an odd variation in the composition of the model wine by evaporation during air blowing cannot be excluded according to literature [38].

Further saturations of the model solutions did not consume oxygen as quickly; therefore, those trials were only monitored for up to 90 days. The tannins’ performance in the red wine was similar to that in the model solution. The tannins consumed total oxygen in the following decreasing order: ellagitannin > grape seed > grape skin > gallotannin. This order is consistent with that reported in the literature—ellagitannins showed the fastest oxygen consumption, followed by the flavanol-based tannins of quebracho (fisetinidin), grape skin and seed, and, finally, gallotannins [39].

In Chianti red wine, which already contains natural polyphenolic compounds, tannins were added at a dosage low enough to avoid the risk of tannin loss by insolubilization/precipitation (100 mg/L), which seems to occur at high dosages [35] and is high enough to be above their sensory threshold [40]. In fact, the astringency of Sangiovese red wine is significantly modified by the addition of 100 mg/L of exotic tannin [15].

**Figure 2:**
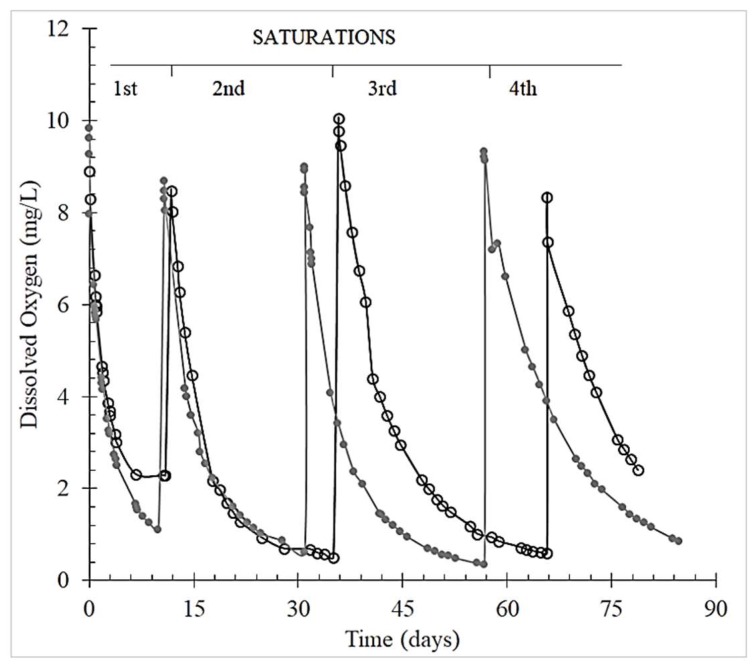
Total Package Oxygen (TPO) consumption by Chianti red wine control (○) and added with grape skin tannin (●) over four subsequent saturations.

Figure 2 exemplifies the O_2_ consumption over time for the Chianti red wine and grape skin tannin: graphs for the other tannins are similar.

The oxygen consumption slowed down after four saturations. In particular, the duration of the TPO consumption ranged from 7 to 14 days for the 1st saturation, 16 to 26 days for the 2nd and 27 to 30 days for the 3rd saturation (Table 1).

Moreover, the total O_2_ consumed by red wine over three saturations was about twice that of the model solution for the grape seed and skin and oak tannins. On average, a TPO of 20.16 mg/L for red wine and 9.21 mg/L for model solution was found, compared to 19.76 mg/L for the control, that is, red wine without added tannins (Table 1). The rate of O_2_ consumption in the red wine always decreased with the addition of tannins—each subsequent saturation cycle took longer to deplete the same amount of O_2_. However, the addition of tannins always reduced the final O_2_ content in red wine to less than that in the control, thus confirming that the extent of oxygen consumption is strongly wine-dependent. Even in the model solutions, the extent to which oxygen was consumed varied greatly, depending on the tannin— which seems to evolve into new compounds with different abilities to take part in redox reactions. In contrast, all red wine samples were able to consume almost all the oxygen over three saturation cycles. As very long time is needed to fully consume oxygen over consecutive wine saturations, the monitoring sometimes focuses on approaching steady state [41,42].

**Figure 3:**
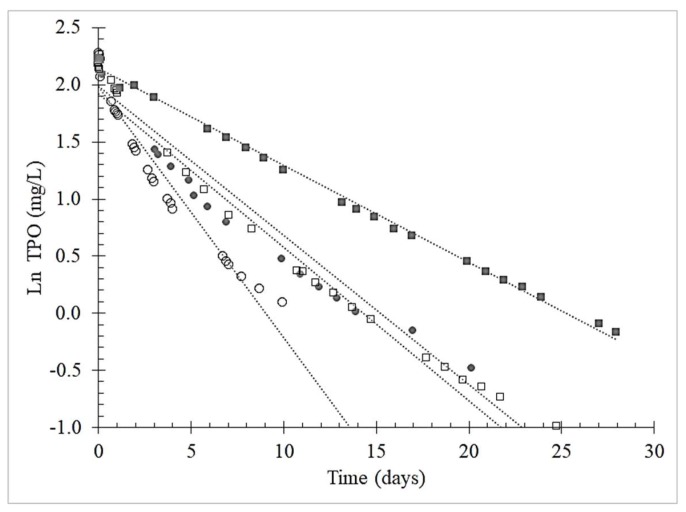
Example of first order kinetic model of Total Package Oxygen (TPO) consumption over four saturation added with oenological grape skins tannins. Legend: (○) first saturation; (●) grape skins tannin; (□) ellagitannin; (■) gallotannin.

The kinetics of oxygen consumption by oenological tannins was modeled based on TPO values over time to find the order of reactions. A first-order kinetic model (Figure 3), with R^2^ > 0.90, fitted the following integral Equation:

Ln[O_2_]_t_ = ‒kt + ln[O_2_]_0_, (1)

where [O_2_]_0_ was the initial TPO concentration after each saturation, [O_2_]_t_ was the TPO concentration at time t and k was the constant rate of oxygen consumption (representing the slope of the kinetic curve). Table 2 shows the kinetic values for the 32 trials; two of them—the red wine control and the red wine with gallotannin—can be improved at R^2^ > 0.90 by fitting with a second-order reaction (1/[O_2_] = A/t + B).

Boulton demonstrated that the time course of oxygen consumption follows either pseudo-first-order or first-order kinetics but only if some of the ferrous ion is quickly returned to the reduced state so that its concentration is essentially constant, rather than declining due to consumption [43]. However, note that the oxygen consumption rate can also follow more complex second-order kinetics, depending on tannin and oxygen concentrations [38]. Previous studies have modeled the oxidative degradation of procyanidins in a model wine system by means of second-order kinetics [44]. Indeed, more complex kinetics were observed when considering the degradation of pentagalloylglucose and ellagic acid-based tannins (hydrolysable) under the same oxidative conditions [45]. In particular, the latter pulse-radiolytic studies revealed that both potential dimerization and disproportionation of the semiquinones are second-order processes. Complexity is related to the extensive formation of oxidative by-products, which may further evolve in secondary reactions, that is, formation of new polymers [46]. In this view, the kinetics of further reactions are most likely affected by the ratio of polymers to monomeric species.

The reaction rate constant k was highest for the 1st saturation in both model solutions and Chianti red wines, confirming that the O_2_ consumption was fastest during the 1st cycle (Table 2). The drop in the OCR of the red wines after each saturation cycle indicates a slowing of the redox reaction in which metals and phenolic substrates take part. On the other hand, the condensed tannins (i.e., from grape seed and skin extracts) in the model wine solution affected the TPO more after the 3rd saturation than after the 2nd saturation; in contrast, the model solutions with the hydrolyzed tannins (i.e., ellagitannins and gallotannins) had similar OCRs after the 2nd and 3rd saturations. This difference is probably due to the chemical structure of the condensed tannins; according to the literature, when oxidation is triggered in a solution containing grape proanthocyanidins, a mixture of new intermolecular and intramolecular bonds can be created. These bonds create different intermediate reaction products, resulting in reduced reactivity of tannin structures. Poncet–Legrand et al. (2010) reported that condensed tannins added in solution at high concentration levels (up to 5 g/L) in an oxidative environment produce further polymerization reactions and a higher ratio extension unit/terminal unit [46]. As a result, during the 3rd saturation the condensed tannins are still involved in the oxygen consumption reaction.

A study of tannins added to a Lambrusco red wine at different concentration levels revealed that all tannins, regardless of their botanical origin, were involved in the production of acetaldehyde, whose concentration was consistently higher in the red wine than in the control wine without added tannins, under the same oxidative conditions. While the acetaldehyde was consumed in 30 days, the color intensity and pigment polymer concentration were higher in the wines with added condensed tannins and ellagitannins than in the controls [47]. This finding is additional confirmation that tannin oxidation following the first saturation cycle can trigger various reaction pathways in red wine, with a progressive reduction in the number of reaction sites available for the consumption of oxygen in subsequent cycles.

In both model solutions and red wines, the fastest OCR was obtained by the addition of oak ellagitannin, probably due to the high number of vicinal ortho –OH groups which can be easily oxidized [48]. While the red wine samples with ellagitannin showed the fastest OCR after the first saturation, in every following saturation the OCR was slower than in the red wine samples with the other tannins. As noted, the oxidation process producing quinones from flavanol-based compounds can be reversed under oenological conditions, regenerating the original substrate and allowing it to be further involved in the redox reaction. Although ellagic acid and its lactones form stable adducts through the involvement of the oxidation products, the model wine solution with ellagitannin consumed oxygen faster than the other model wines in every saturation cycle—not just the first. This continued oxygen consumption could be due to the lack of nucleophilic wine compounds such as flavanols, ethanol, anthocyanins and thiols that decrease tannin reactivity [49].

Gallotannin slowed down the oxygen consumption rate after the first saturation in both the red wine and the model solution; these findings are consistent with the previous report by Pascual et al. [39]. As can be seen in Table 2, the reaction constant rate k of the red wine with gallotannins was higher after the 2nd and 4th saturations than after the 1st and 3rd saturations. This contrasts with the other oenological tannin reaction rates, which decreased after each wine saturation. The phenomenon of quinone–phenol dimerization via coupled oxidation can explain these findings, since the resulting dimers have lower redox potentials than the initial phenols with two important implications in the oxygen consumption mechanisms in wine: dimers are more readily oxidized (more reactive to oxygen than the original phenol) and if the original phenol is oxidized to its quinone, the dimer hydroquinone can reduce it restoring the original compound [21]. Furthermore, flavonoids, semiquinones and quinones also seem able to coordinate metals (Fe, Cu), albeit usually with low affinity at wine pH. In particular, over the pH range 2.0 to 4.5, the green iron(II) semiquinone complex is dominant, while the coordinated iron(II) prevents the semiquinone from undergoing disproportionation to catechol and benzoquinone [50]. However, having the ability to chelate metals is not equivalent to having a significant antioxidant action. In fact, flavonoid–metal ligand could favor the removal of the metal from the reaction media depriving the reaction from a catalyst but could also remain in the reaction milieu. In the latter case, to operate as an antioxidant the flavonoid–metal complex has to be less efficient compared with the ‘‘free” metal as catalyst of free radical formation.

Note, though, that the ability to coordinate metals has an antioxidant effect only under certain conditions: for example, if a flavonoid binds with a metal to create a compound that is less oxidizing than the metal itself, the metal’s catalytic effect is reduced/eliminated and fewer free radicals are formed [51].

The reduction potential of the Fe(III)/Fe(II) redox couple is about ∼350 mV under wine conditions [52], so the chelates with iron(II), by stabilizing Fe(II) relative to Fe(III), are expected to increase the reduction potential of the Fe(III)/Fe(II) couple, thereby making the oxidation of Fe(II) more thermodynamically unfavorable.

In both the model solution and the red wine, the grape seed tannin increased the oxygen consumption faster than skin tannin in the first two saturations (Table 2). This kinetic trend is possibly due to the different chemical structures and different mean degrees of polymerization (mDP) of these two condensed tannins. It is well known that seed tannins are predominantly composed of procyanidins with a lower mDP—around 10—compared to skin tannins, as well as containing high levels of monomeric flavan-3-ols and oligomers, which are readily oxidizable, as the main terminal units. In contrast, skin tannins have a higher mDP—around 30—and consist of procyanidins, prodelphinidins and increasing gallated/galloylated terminal units, which are less oxidizable [53,54].

However, in subsequent saturation cycles, the skin tannin boosted the consumption of oxygen more than the seed tannin, in both the model and red wines. Previous studies suggested that depolymerization of long chain condensed tannins in wines (mainly derived from grape skin) might occur in wines as a consequence of oxygen consumption cycles and that OCR observed in the subsequent cycles are strongly affected by the nature of the depolymerization products; in particular the depolymerization pattern involving the release of epicatechin 3-O-gallate and the increase in catechin as terminal units accelerates the rate of the oxygen consumption reaction [4].

## 3. Materials and Methods

### 3.1. Oenological Tannins

Four commercial oenological tannins were studied: (i) tannin from red grape seeds, (ii) tannin from grape skins, (iii) ellagitannin (from American oak) and (iv) gallotannin (from nutgalls); oenological tannins used are new formulations and the commercial brand is restricted by the Companies. Essential compositional information is provided to support the scientific findings (Table 3). Each tannin was added to the model wine solution and the red wine—levels reported in Table 3. The type of tannin and the dosage were selected in accordance with common winemaking practices. In particular, gallotannins and quercus hydrolysable tannins contains 12% and 27% of tannins, respectively [14].

Samples were analyzed for total (iron reactive) polyphenols and tannins using the method of Harbertson et al. [55] which is based (i) on the ability of protein (i.e., bovine serum albumin, BSA) to precipitate tannins and (ii) on the reactivity of ferric chloride with phenolic compounds that possess ortho-dihydroxyl groups, as previously described [56]. Briefly, the wine tannin are precipitated with bovine serum albumin (BSA), then the pellet is dissolved in buffer and the tannin are determined by reaction with ferric chloride, yielding a colored product quantified at 510 nm (UV–Vis spectrophotometer Cary 60, Agilent Technologies, Santa Clara, CA) and using (+)-catechin as calibration standard (mg/L CE) (Sigma, Milano, Italy).

### 3.2. Model Solution and Red Wine

The model wine solution was prepared with ethanol (12% v/v) and tartaric acid (2.5 g/L) purchased from Enartis (Florence, Italy). Approximately 5 mg/L of iron(II) and 0.5 mg/L copper(II) were added to reproduce the typical catalytic conditions expected in wine. The pH was adjusted to 3.6 with sodium hydroxide (1 M) and hydrochloric acid (1 M) supplied by Sigma Aldrich Laborchemikalien GmbH (Seelze, Germany). The Chianti red wine, vintage 2017, was a Designation of Origin Product (from Ruffino, Tuscany, Italy) with the following composition: pH 3.55, total polyphenolic compounds 2458 mg/L as catechin equivalent, free sulfur dioxide 27 mg/L (total sulfur dioxide 107 mg/L), iron 3 mg/L and copper 0.1 mg/L.

### 3.3. Oxygen Measurements

The oxygen level in each sample was measured with the NomaSense P300 oxygen analyzer (Nomacorc, Thimister Clermont, Belgium), based on non-invasive oxy-luminescence technology. Both headspace (HSO) and dissolved oxygen were analyzed using 5-mm diameter oxy-luminescence dots PSt3 (Nomacorc, Thimister Clermont, Belgium) placed inside standard 0.375 L transparent glass bottles (Zignago Vetro, Portogruaro, Italy). Total package oxygen (TPO) was the sum of dissolved oxygen (DO) in the wine plus oxygen in the headspace (HSO) after bottling [57]. The synthetic cork “Select Green 100” (Nomacorc, Thimister Clermont, Belgium) was chosen for capping due to its consistently low oxygen transmission rate (OTR), ensuring a negligible contribution to oxygen levels during the trials with 0.4 mg of O_2_ after 3 months.

### 3.4. O_2_ Saturation Method

Up to four cycles of O_2_ saturation and consumption were performed. All the samples (model solution and red wine) were saturated by racking under air exposure until the dissolved oxygen concentration reached a stable plateau. The samples were then poured into 0.375-L transparent glass bottles containing oxy-luminescent dots. In order to minimize HSO, nitrogen was blown into the headspace for one minute before capping. The oxygen consumption was monitored using an optical fiber until approaching a steady state, then the bottles were uncorked and further oxygen saturations were applied by inserting a long narrow glass tube into the bottle and gently blowing air, before recorking as previously described. Care was taken to ensure no liquid was lost and that the HSO was kept constant for the whole duration of the trial.

The two saturation procedures herein used provided similar results in terms of time required and final DO level. The empirical criteria were followed to consider a saturation process to be complete based on the stable level of DO in wine. A similar approach was applied at industrial scale already [28]. Although some unexpected variability on O_2_ saturation level occurred in successive saturation processes, the global impact on the current findings is limited at winemaking level. Singleton reported approximate values of solubility of oxygen from air into wine saturated at room temperature, indicating roughly 6 mL/L or 8 mg/L at the atmospheric pressure [58]. In further studies after Boulton et al. it was confirmed that the oxygen solubility in wine should rise to 40 mg/L if a pure oxygen headspace is used [21] and the O_2_ measurement is affected by alcohol, sugars and phenolics content as well [38].

### 3.5. Data Storage and Processing

XLStat-premium 2018.3 for Excel (Addinsoft, Paris, France) was used to store the oxygen measurement data and elaborate the kinetic curves to gain insight into the possible mechanism of reaction pathways.

The drop in the concentration of O_2_ over time (t) can be written as:

‒d[O_2_]/dt = k [O_2_]. (2)

Rearrangement yields the following:

d[O_2_]/[O_2_] = ‒k dt. (3)

Integration yields:

ln [O_2_] = ‒kt + C. (4)

When t = 0, [O_2_] = [O_2_]_0_. [O_2_]_0_ is the original starting concentration of O_2_.

Substituting into the equation, we obtain:

ln [O_2_]_0_ = ‒k (0) + C; therefore, C = ln [O_2_]_0._ (5)

The integrated form for first-order kinetics can now be written as follows:

ln [O_2_]_t_ = ‒kt + ln [O_2_]_0._ (6)

The integrated first-order equation is the equation of a straight line in which the y-value is ln [O_2_], the slope equals negative k, the x-value is t and the y-intercept is ln [O_2_]_0_.

## 4. Conclusions

The addition of ellagitannin to red wine increased the rate of O_2_ consumption, providing an effective tool in winemaking operations characterized by high levels of O_2_ uptake—when it is important to have instant protection against oxidation. Although the ellagitannin may have great potential as an antioxidant in wine, its effect on O_2_ consumption tends to drop rapidly with time; therefore, it has only a limited application. In contrast, although the skin tannins showed more consistent reactivity and lower OCR in each saturation when added to red wine, the fact that grape seed tannins are typically higher in reactive flavonoids makes them more suitable than ellagitannin for guaranteeing fast oxygen consumption. The kinetics of oxygen consumption after the addition of gallotannin demonstrated low performance in terms of protection against oxygen exposure; nevertheless, this tannin might be considered to clarify wines and musts, to chelate and remove catalytic metals and to protect grapes and musts from enzymatic oxidation. The relevance of such findings to winemaking is likely to be considerable.

## Figures and Tables

**Table 1:** Oxygen consumption parameters in model wines and Chianti red wines with tannins added. Codification of wines/tannins are detailed in Table 3.

Sample Code	Duration of O_2_ Consumption (days)			TPO Consumed (mg/L)			Final O_2_ Level (mg/L)			Total O_2_ Consumed (mg/L)
	1^st^ sat.	2^nd^ sat.	3^rd^ sat.	1^st^ sat.	2^nd^ sat.	3^rd^ sat.	1^st^ sat.	2^nd^ sat.	3^rd^ sat.	
MWse	43	29	14	7.91	1.24	1.51	0.87	7.55	7.71	10.08
MWsk	43	26	14	7.65	2.20	2.40	1.05	6.86	8.81	8.42
MWet	43	29	14	6.96	3.29	1.88	0.33	4.97	6.83	11.86
MWgt	30	29	14	3.10	2.51	1.06	4.94	4.16	8.27	6.68
Control	7	16	27	6.57	13.3	9.37	2.31	0.70	0.67	19.76
CHse	10	18	27	8.57	8.03	8.64	0.89	0.67	0.58	20.32
CHsk	10	20	26	8.72	8.06	8.65	1.10	0.16	0.34	20.37
CHet	10	26	28	7.92	9.11	9.13	0.43	0.46	0.56	20.33
CHgt	14	22	30	6.70	7.71	7.84	1.80	0.45	0.74	19.61

sat. = saturation.

**Table 2:** First-order kinetic equations for the consumption of oxygen on model solutions and wines.

Experiment	1^st^ Saturation	2^nd^ Saturation	3^rd^ Saturation	4^th^ Saturation
MWse	y = −0.053x + 2.107 (R^2^ = 0.99)	y = −0.005x + 2.159 (R^2^ = 0.97)	y = −0.012x + 2.218 (R^2^ = 0.97)	
MWsk	y = −0.049x + 1.947 (R^2^ = 0.98)	y = −0.011x + 2.177 (R^2^ = 0.95)	y = −0.020x + 2.343 (R^2^ = 0.99)	
MWet	y = −0.071x + 1.715 (R^2^ = 0.98)	y = −0.017x + 2.046 (R^2^= 0.94)	y = −0.019x + 2.194 (R^2^ = 0.98)	
MWgt	y = −0.016x + 1.984 (R^2^ = 0.93)	y = −0.011x + 2.242 (R^2^ = 0.99)	y = −0.010x + 2.255 (R^2^ = 0.96)	
Control wine	y = −0.133x + 1.857 (R^2^ = 0.77)	y = −0.119x + 1.792 (R^2^ = 0.92)	Y = −0.096x + 2.087 (R^2^ = 0.96)	y = −0.091x + 2.047 (R^2^ = 0.99)
CHse	y = −0.242x + 1.911 (R^2^ = 0.94)	y = −0.148x + 1.954 (R^2^ = 0.97)	y = −0.102x + 1.902 (R^2^ = 0.96)	y = −0.077x + 2.030 (R^2^ = 0.96)
CHsk	y = −0.218x +1.982(R^2^ = 0.95)	y = −0.134x + 1.916 (R^2^ = 0.96)	y = −0.131x + 1.987 (R^2^ = 0.98)	y = −0.078x + 2.090 (R^2^ = 0.98)
CHet	y = −0.307x + 1.896 (R^2^ = 0.98)	y = −0.121x + 1.912(R^2^= 0.94)	y = −0.102x + 2.131 (R^2^ = 0.99)	y = −0.071x + 2.085 (R^2^ = 0.99)
CHgt	y = −0.105x+ 1.724 (R^2^ = 0.77)	y = −0.133x + 1.854 (R^2^ = 0.97)	y = −0.081x + 1.903 (R^2^ = 0.96)	y = −0.087x + 2.207 (R^2^ = 0.99)

**Table 3:** Tannins and dosages used in the experiment.

Sample Code	Sample Composition	Tannin Composition
MVse	Model wine solution + seed tannin 1 g/L	Seed tannin: 733 mg TPC/L of which 188 mg tannins/L (as CE)
MVsk	Model wine solution + skin tannin 1 g/L	Skin tannin: 856 mg TPC/L of which 172 mg tannins/L (as CE)
MVet	Model wine solution + ellagitannin 1 g/L	Ellagitannin: 478 mg TPC/L of which 53 mg tannins/L (as CE)
MVgt	Model wine solution + gallotannin 1 g/L	Gallotannin: 877 mg TPC/L of which 404 mg tannins/L (as CE)
CH	Chianti red wine (control)	total polyphenolic compounds (TPC) 2458 mg/L as catechin equivalent (as CE)
CHse	Chianti red wine + seed tannin 0.1 g/L	Tannin as above
CHsk	Chianti red wine + skin tannin 0.1 g/L	Tannin as above
CHet	Chianti red wine + ellagitannin 0.1 g/L	Tannin as above
CHgt	Chianti red wine + gallotannin 0.1 g/L	Tannin as above
